# Design of intrinsically stretchable and highly conductive polymers for fully stretchable electrochromic devices

**DOI:** 10.1038/s41598-020-73259-x

**Published:** 2020-10-05

**Authors:** Youngno Kim, Chanil Park, Soeun Im, Jung Hyun Kim

**Affiliations:** Department of Chemical and Biomolecular Engineering, Yonsei University, Seoul, 03722 South Korea

**Keywords:** Energy science and technology, Materials science, Nanoscience and technology

## Abstract

Stretchable materials are essential for next generation wearable and stretchable electronic devices. Intrinsically stretchable and highly conductive polymers (termed ISHCP) are designed with semi interpenetrating polymer networks (semi-IPN) that enable polymers to be simultaneously applied to transparent electrodes and electrochromic materials. Through a facile method of acid-catalyzed polymer condensation reaction, optimized ISHCP films show the highest electrical conductivity, 1406 S/cm, at a 20% stretched state. Without the blending of any other elastomeric matrix, ISHCP maintains its initial electrical properties under a cyclic stretch-release of over 50% strain. A fully stretchable electrochromic device based on ISHCP is fabricated and shows a performance of 47.7% ∆T and high coloration efficiency of 434.1 cm^2^/C at 590 nm. The device remains at 45.2% ∆T after 50% strain stretching. A simple patterned electrolyte layer on a stretchable electrochromic device is also realized. The fabricated device, consisting of all-plastic, can be applied by a solution process for large scale production. The ISHCP reveals its potential application in stretchable electrochromic devices and satisfies the requirements for next-generation stretchable electronics.

## Introduction

In academic and industrial communities, the interest of stretchable electronics has increased. Deformable properties are in demand for stretchable devices, which include light-emitting diodes, batteries, artificial electronic skin (E-skin), supercapacitors, sensors, transistors, electrochromic devices (ECDs), and more^1–4^. Among these devices, ECDs have been anticipated for promising applications in optoelectronics due to their low power consumption (energy cost savings) and the low cost of their basic materials^5^.

The attractiveness of stretchable ECDs is in the possibility of making wearable devices and displays, among other potential applications. Zhao et al*.* showed large color changing area in the visible light range with photonic crystal prepared by nano-imprinting on PDMS. It indicated the potential for applications of camouflage functional devices^6^. Liu et al. presented a sandwiched tungsten trioxide/silver nanotrough network/PEDOT:PSS multi-layer transparent electrode with excellent conductivity and transparency^7^. This hybrid electrode provided new opportunities for wearable electronics applied on elastomeric substrates. Like these prior researches, stretchable ECDs segue into the next generation of ECDs. There have been various reports on the development of good-performance stretchable ECDs for wearable electronics, but these previous studies have been limited to a stretchable electrode and gel-type electrolytes^8–10^. Design of wavy or pre-strained geometry and conductors embedded in a stretchable matrix, such as Ag nanowire-embedded polydimethylsiloxane (PDMS), PEDOT:PSS/PU and CNT/PDMS, were attempted for stretchable electrode materials but the conductors did not have their own intrinsic deformability^10,11^. Especially, the composites of conductor embedded in elastomer matrix must have reduction of electrical properties on stretched by percolation threshold point. It is difficult to achieve high deformability of electronic devices because of elastomer matrix that is not electrically conductive. These approaches have limitations that included complicated processing or difficulty in achieving high device density. In the case of non-electrode materials, hydrogels are so deformable and elastic that electrochromic materials embedded in hydrogel electrolyte were attempted in many previous studies^12,13^.

ECDs require two electrodes (anode and cathode) made of electrochromic materials and electrolytes. At least one of the two electrodes is coated with an electrochromic (EC) material that is an anodically or cathodically coloring electrochrome. Without suitable stretchable electrochromic materials, even a good electrolyte cannot operate well. Therefore, it is necessary to establish intrinsically stretchable electrochromic materials. Intrinsically stretchable electrochromic materials would realize ideal stretchable ECDs through a facile fabrication process and high coloration efficiency.

For intrinsically stretchable conjugated polymers, in our previous work, the copolymerization of soft template was investigated^13^. However, it was narrowly able to be used as stretchable interconnectors because it had limitations of low electrical conductivity as an electrode material and a relatively complicated copolymerization process. In this work, we designed an intrinsically stretchable and highly conductive polymer (ISHCP) as a novel stretchable electrode and electrochromic material for a facile fabrication method and high conductivity. We did not focus on extreme mechanical stretchability and highest electrical conductivity, but instead on the intrinsic deformability and suited applicability for fully stretchable all-plastic electrochromic devices. The optimized ISHCP material has several advantages in simplified device configuration: high coloration efficiency, low power consumption, and high stretchability.

## Results and discussion

**Figure 1:**
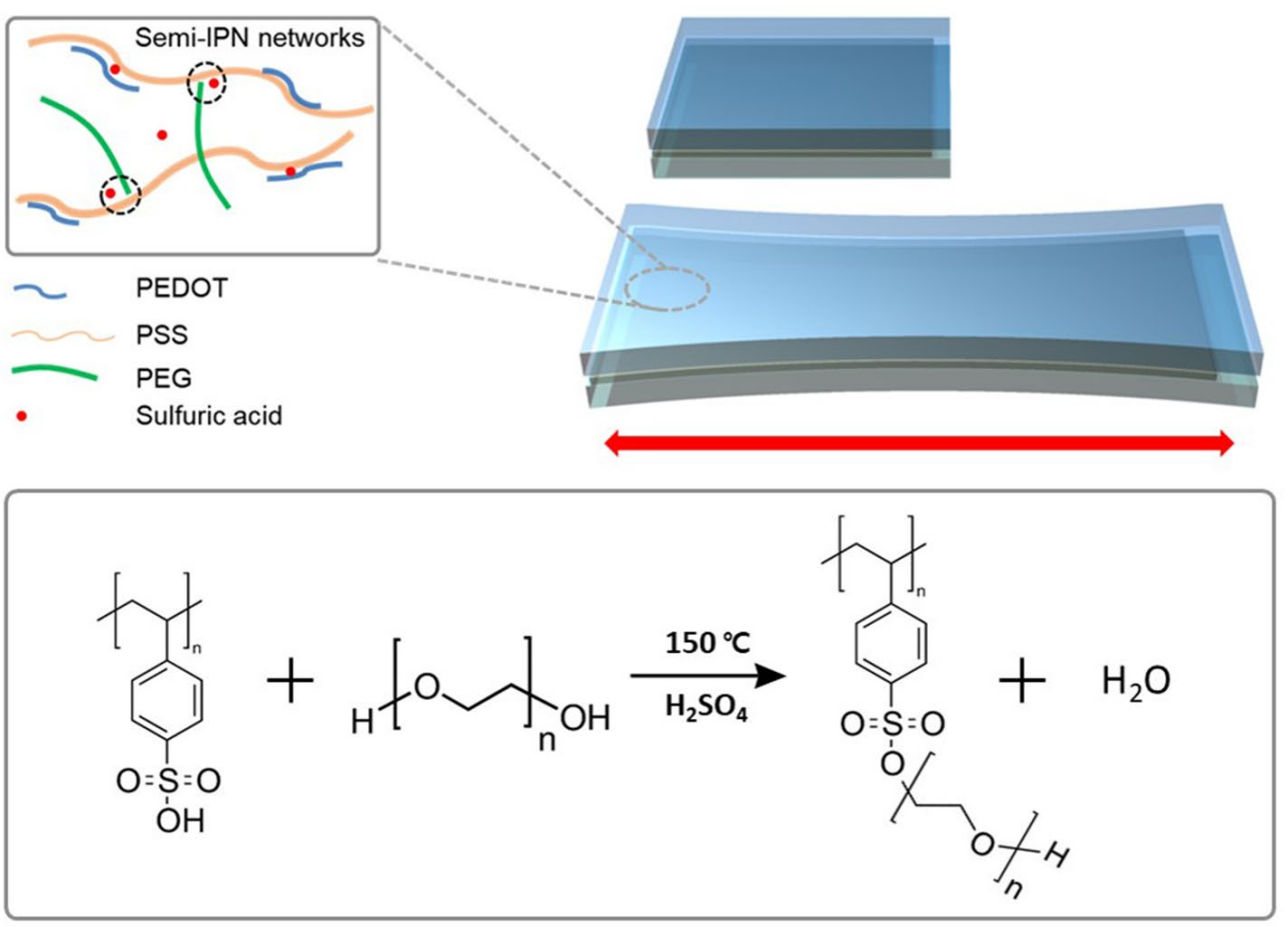
Semi-interpenetrating networks of ISHCP films and polycondensation reaction between polystyrene sulfonate and polyethylene glycol.

Conjugated polymers, as representative organic electrochromic materials, do not intrinsically have good stretchable properties because of their rigid chemical structures^14^. In this work, we designed ISHCP based on semi-interpenetrating networks (semi-IPN) indicating a polymer that has interlaced networks on a polymer scale. The networks included bulky structures and dynamic bonds such as a hydrogen bonding can enhance the stretchability of ISHCP thin films. ISHCP was able to perform as the electrode and electrochromic layers simultaneously. The fabrication of semi-IPN through the polycondensation reaction between hydroxyl groups of polyethylene glycol (PEG) and polystyrene sulfonic acid (PSS) under sulfuric acid catalyst and optimized temperature is shown in Fig. 1.

The ductile and stretchable properties, changed by semi-IPN, was focused on during thermal annealing of the ISHCP thin films. The effects that increase the stretchability in combination with PEG were confirmed through a comparison of neat PEDOT:PSS and H_2_SO_4_ in-situ polymerized PEDOT:PSS. The forces on stretching of ISHCP films could dissipate due to its bulky structures with semi-IPN and efficient non-covalent bonds^15^. To enhance the stretchability of ISHCP materials, the PEG was selected as a soft segment whose chemical structure can be easily rotated because of its ether group. The specific molecular weight of PEG was optimized by resistance change as a function of strain in ISHCP coated on an elastomeric styrene-ethylene-butadiene-styrene (SEBS) substrate. The SEBS was selected as the substrate for fabrication of ISHCP thin films due to its excellent transparency, stretchability and elasticity. Figure S2 shows that the transmittance of SEBS with thickness 100 µm was 90.4% at wavelength 550 nm.

**Figure 2:**
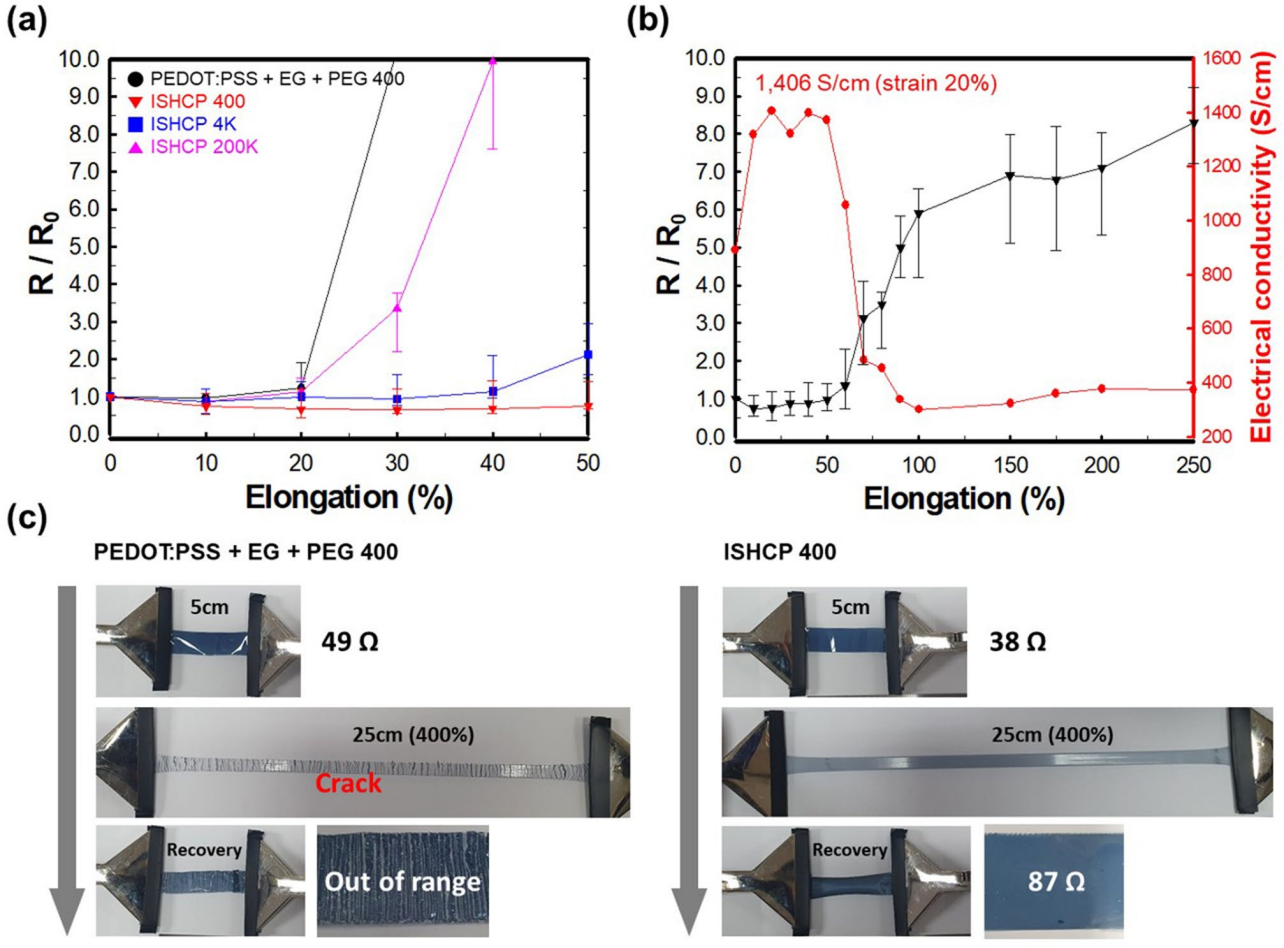
**(a)** Change of relative resistance (R/R_0_) of ISHCP, with different conditions, coated on SEBS substrate under 0 to 50% strain. **(b)** Variation of the relative resistance and electrical conductivities of optimized ISHCP coated on SEBS substrate as a function of the strain. **(c)** Demonstration of deformable ISHCP coated on a SEBS substrate with stretch-release under 400% strain.

PEG with varying molecular weight, from 400 to 200,000, was added to the PEDOT:PSS solution. Molecular weights of more than 200,000 were excluded because PEG with high molecular weight did not dissolve in the water. All solutions were coated with an identical wet thickness by a bar coater and with the same solute ratio. The change in electrical resistance of the fabricated thin films coated on the SEBS substrate was measured under strain (Fig. 2a). The electrical properties of PEDOT:PSS, which was mixed with ethylene glycol (EG) and PEG-400, was drastically decreased up to 20% strain. However, in case of ISHCP films with different molecular weights of PEG, ISHCP-400 (This means that the molecular weight of the added PEG is 400) maintained its initial electrical properties under 50% strain. The hydroxyl group of PEG formed a covalent bond with the sulfate of PSS by H_2_SO_4_ catalyst, and bulky dynamic networks dissipated the force when stretched^16^.

Although PEG is water-soluble and soft polymer, high molecular weight PEG blending with PEDOT:PSS can decrease the electrical conductivity because of its insulating properties^17^. As shown in Table S1, ISHCP-400 has the highest electrical conductivity among different conditions. Through H_2_SO_4_ in-situ polymerization of PEDOT:PSS, the electrical conductivity was improved from 258 to 891 S/cm with the same recipe (7 wt% EG and 1 wt% PEG-400). The sulfuric acid induced a crystalline nanofibril structure of PEDOT chains, which segregate the positively charged PEDOT and negatively charged PSS^18^. The difference in dry thickness, even though coated with the same wet thickness, is due to the increased viscosity of the coating solution as the molecular weight of PEG increased. However, ISHCP-400 films coated on a SEBS substrate via the optimal recipe had the lowest sheet resistance. Because the molecular chain was aligned unidirectionally under 20% strain, ISHCP-400 showed the highest electrical conductivity of 1406 S/cm shown in Fig. 2b. In order to confirm the aligned PEDOT chain on stretched state, the polarized UV–Vis-NIR spectra was measured under tensile strains with parallel and perpendicular with respect to the stretching direction (Fig. S3). In case of unstretched ISHCP-400, the absorption spectra showed the same tendency regardless of the angle between the polarized incident light and stretching direction. However, the 20 and 50% unidirectionally stretched ISHCP-400 films showed the distinct difference at NIR, which indicates the bipolaron state of PEDOT chain. It suggested that the anisotropy of PEDOT chains upon stretched state improved the electrical properties of ISHCP-400 thin films.

With more than 60% strain, the electrical conductivity of the optimized ISHCP on a SEBS substrate was decreased but maintained reasonable electrical properties of about 400 S/cm at 250% strain. Even when extending the sample up to 250%, the electrical conductivity was retained, but the value was not taken into account because it was too uneven, and the deviation was significant. In particular, depending on the presence or absence of semi-IPN, the stretchability differed significantly. As shown in Fig. 2c, The distinctive fracture occurred in PEDOT:PSS films without semi-IPN, and its electrical property was entirely lost under 400% strain. However, ISHCP-400 films coated on SEBS substrates showed minimal change in resistance, from 39 to 87 Ω, under 400% large deformation. Table S2 shows the deformability of transparent electrode and electronic device in recent literatures. Compared to other works, ISHCP-400 presented superior deformability at 50% strain. It could be simply used as electrodes and electrochromic materials, while other studies had complex design of structure such as organic/inorganic hybrid system. It was possible to operate the ECD on up to 100% strain due to this simple structure.

**Figure 3:**
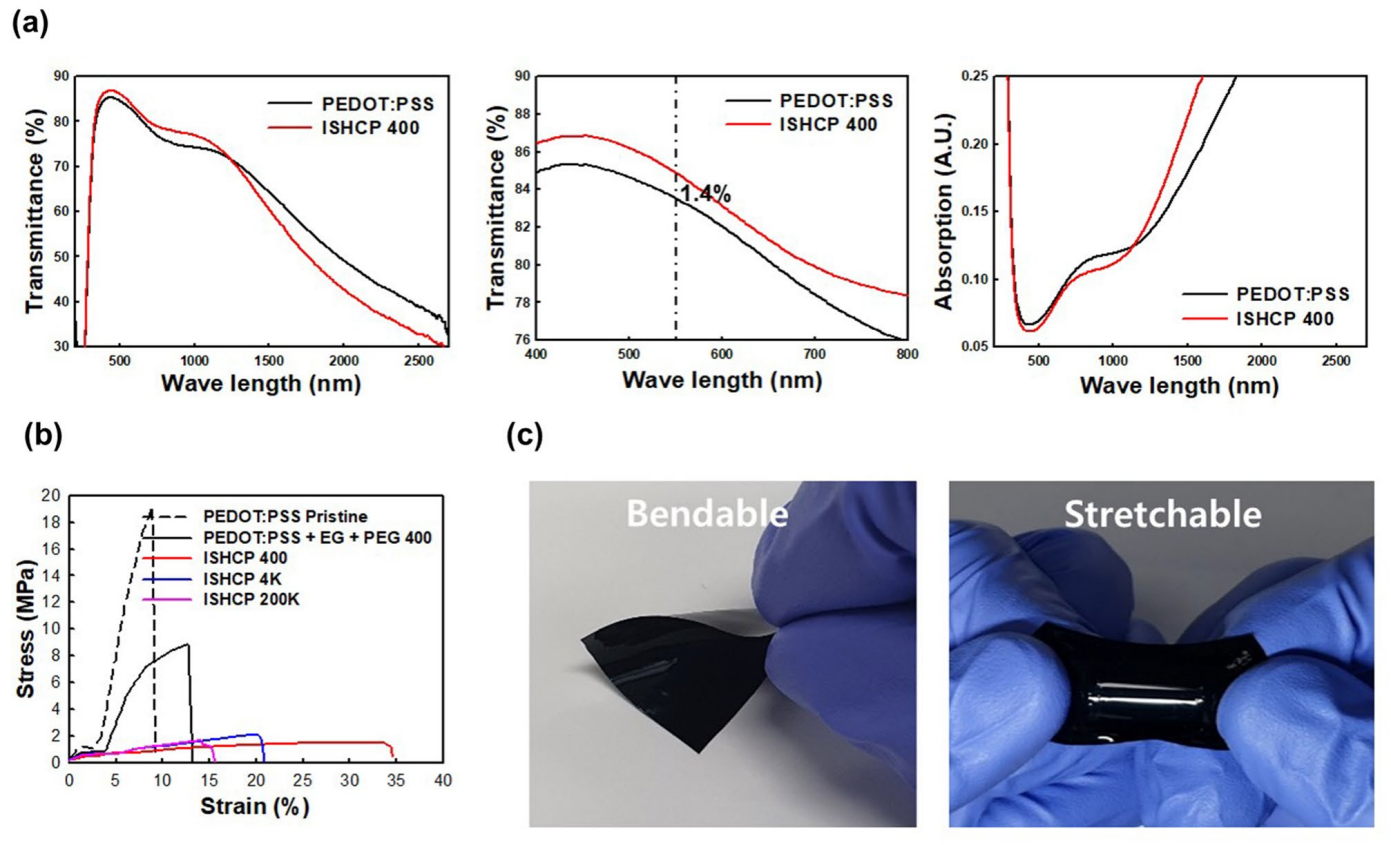
**(a)** Transmittance (left and middle) and absorption (right) spectra measured with a wavelength range of 200–2700 nm for PEDOT:PSS and ISHCP-400 as a function of additional doping by H_2_SO_4_. **(b)** Stress–strain curves of freestanding PEDOT:PSS and ISHCP sheets as a function of H_2_SO_4_ and molecular weight of PEG. **(c)** Images of bendable and stretchable freestanding ISHCP-400 sheets without a substrate.

This report showed an improvement in chemical doping level and mechanical stretchability through H_2_SO_4_ in-situ polymerization and combination with PEG. The simple acid-catalyzed condensation could be used to design intrinsically stretchable and conductive polymers without the introduction of complicated copolymerization or tangled geometric design, such as a pre-strained system and Kirigami structures^19^. UV–Vis-NIR absorbance spectroscopy has been used to investigate the oxidation level of PEDOT chains with H_2_SO_4_ doping (Fig. 3a). Neat PEDOT:PSS has a substantial doping level because of PSSA, but the absorption peak around the visible light region (attributed to neutral chains) and above 1250 nm (attributed to bipolarons, i.e., the dicationic form) were reversed by additional H_2_SO_4_ chemical doping^20^. Therefore, its potential as a transparent electrode was confirmed by a 1.4% difference in transmittance at 550 nm. The interpenetrating structure, which was formed by acid catalyzed polycondensation, was verified by ATR-FTIR spectra from freestanding sheets of PEDOT:PSS and ISHCP-400 shown in Fig. S1. The absorption peak at 1366 cm^−1^, assigned to hydroxyl group in-plane bending, decreased in ISHCP films because of polycondensation between PSS and PEG.

Additionally, the mechanical properties under various recipes were determined using tensile testing of freestanding sheets (Fig. 3b). To measure the own properties of PEDOT:PSS and ISHCP without the elastomeric SEBS substrate, the freestanding sheets were obtained by casting and peeling off the petri dish. The freestanding sheets of neat PEDOT:PSS showed a high Young’s modulus and fracture at 9% strain because it has no elasticity^21^. When a soft segment was included, such as EG and PEG, the fracture point was slightly increased, but the Young’s modulus was still high. In comparison, the ISHCP films with semi-IPN based on H_2_SO_4_ in-situ polymerized PEDOT:PSS showed a dramatically decreased Yong’s modulus (two orders of magnitude) and deformability at a maximum of 35% strain. Freestanding ISHCP-400 sheet was the best performing and showed the same tendency when coated on elastomeric SEBS substrates. Images showing the bendable and stretchable optimized freestanding ISHCP-400 sheets are shown in Fig. 3c.

**Figure 4:**
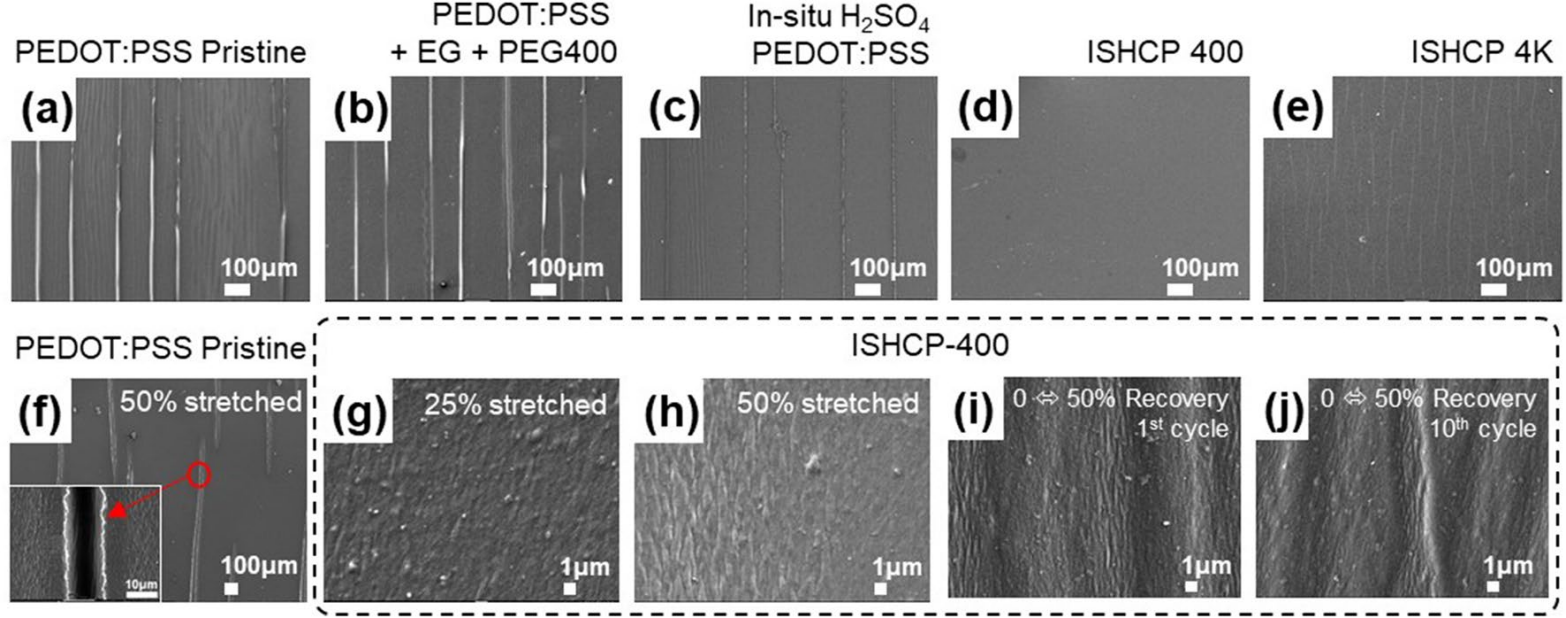
SEM images of PEDOT:PSS and ISHCP coated on SEBS substrates at the **(a–e)** 1st stretch-release cycle and at **(f–j)** different stretching conditions.

Micro-scale scanning electron microscope (SEM) images were collected on the surface of neat PEDOT:PSS and ISHCP films coated on SEBS substrates after one stretch-release cycle from 0 to 50% strain (Fig. 4a–e). Cracks were plainly observed in all cases except for ISHCP-400. The added PEG with various molecular weight to improve the stretchability was controlled by the same mass. The active numbers of hydroxyl end groups of PEG 400 were more than PEG 4K at the same mass basis. Thus, the interlaced networks by polycondensation reaction of ISHCP-400 were effectively able to dissipate the force upon stretching. This phenomenon shows that ISHCP-400 maintained almost 100% of its original electrical properties and dissipate the force generated under strain, as shown earlier in Fig. 2a. On the other hand, the neat PEDOT:PSS sample cracked, and the charge could not be transferred by hopping the 10 µm range of the crack when stretched with a 50% strain. The electrical flow was completely cut-off (Fig. 4f).

To obtain more detailed information, the ISHCP-400 film was measured via SEM at the 1 µm scale while stretched with 25 and 50% strain. An obvious rupture or cut-off area was not discovered on the films though some wrinkles were present when restored to its initial state (Fig. 4g–j). It was confirmed that the shape of the wrinkles became more distinct from the 1st to the 10th cycle. The wrinkles that occurred at the beginning of the stretch-release cycling improve the elasticity of ISHCP-400 films.

**Figure 5:**
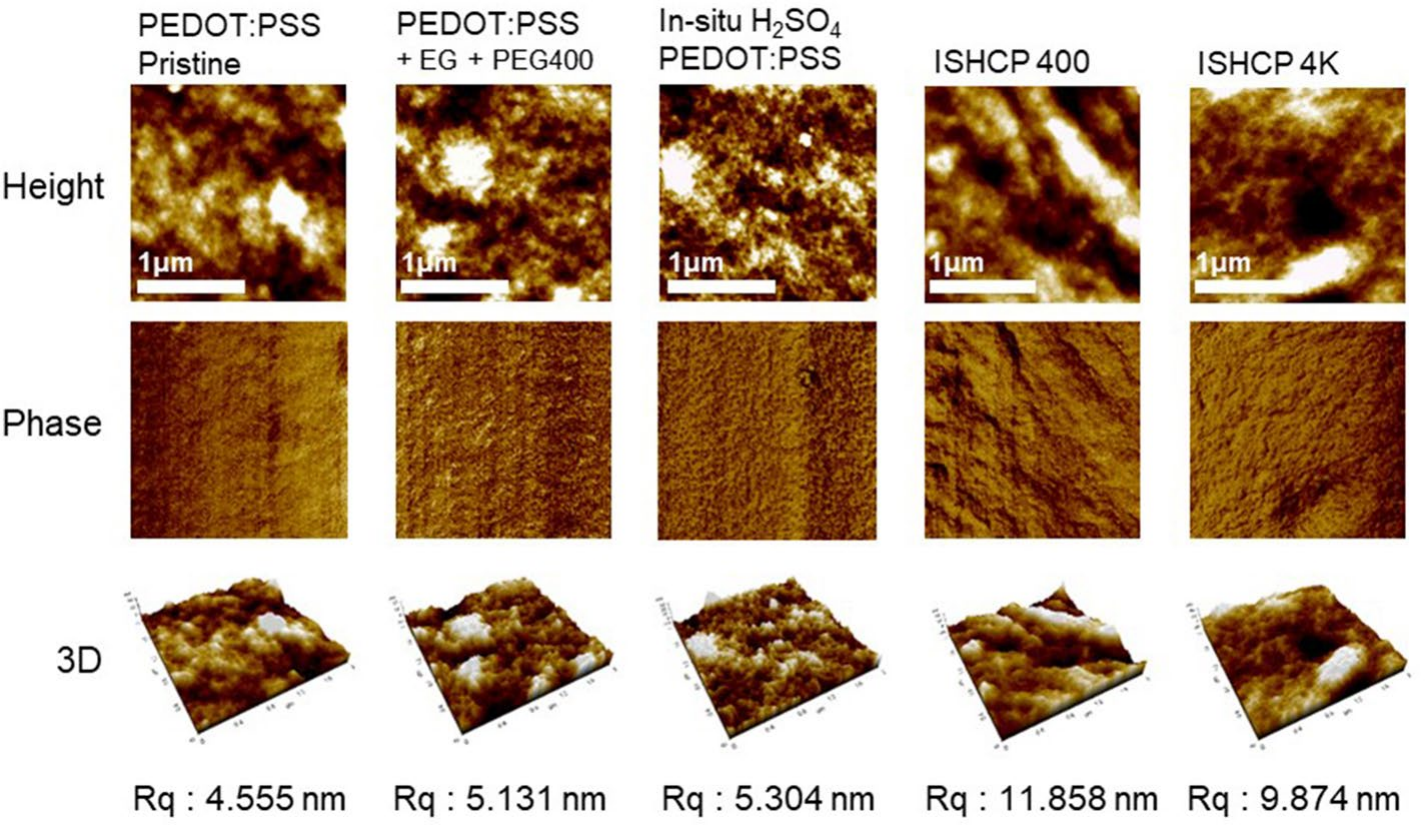
AFM topographic images (top), phase images (middle), and 3D images (bottom) of PEDOT:PSS and ISHCP with different conditions coated on SEBS substrates.

H_2_SO_4_ or EG is commonly used as an additive that induces phase separation between PEDOT and PSS^22,23^. When the additives are mixed with PEDOT:PSS, the size of the PEDOT increased as it is aggregated and becomes highly electrically conductive^24–26^. In atomic force microscope (AFM) images, it was confirmed in ISHCP-400 that the shape of PEDOT grain was different from other samples, and a linear-like nanofibril acted to improve the connection with PEDOT chains under strain. On the other hand, ISHCP-4K showed a similar tendency, but the electrical connection was interrupted because of insulating and long PEG chains. As shown in the right side of Fig. 5, The effect of PEG-4K on the relationship between electrical properties and mechanical stretchability can not be expected because of a long distance between PEDOT grains. The film roughness (R_q_) of ISHCP-400 was 11.858 nm, 2.6 times the pristine PEDOT:PSS due to aggregation of PEDOT grains. This wrinkled surface can increase the deformability of films^27^.

**Figure 6:**
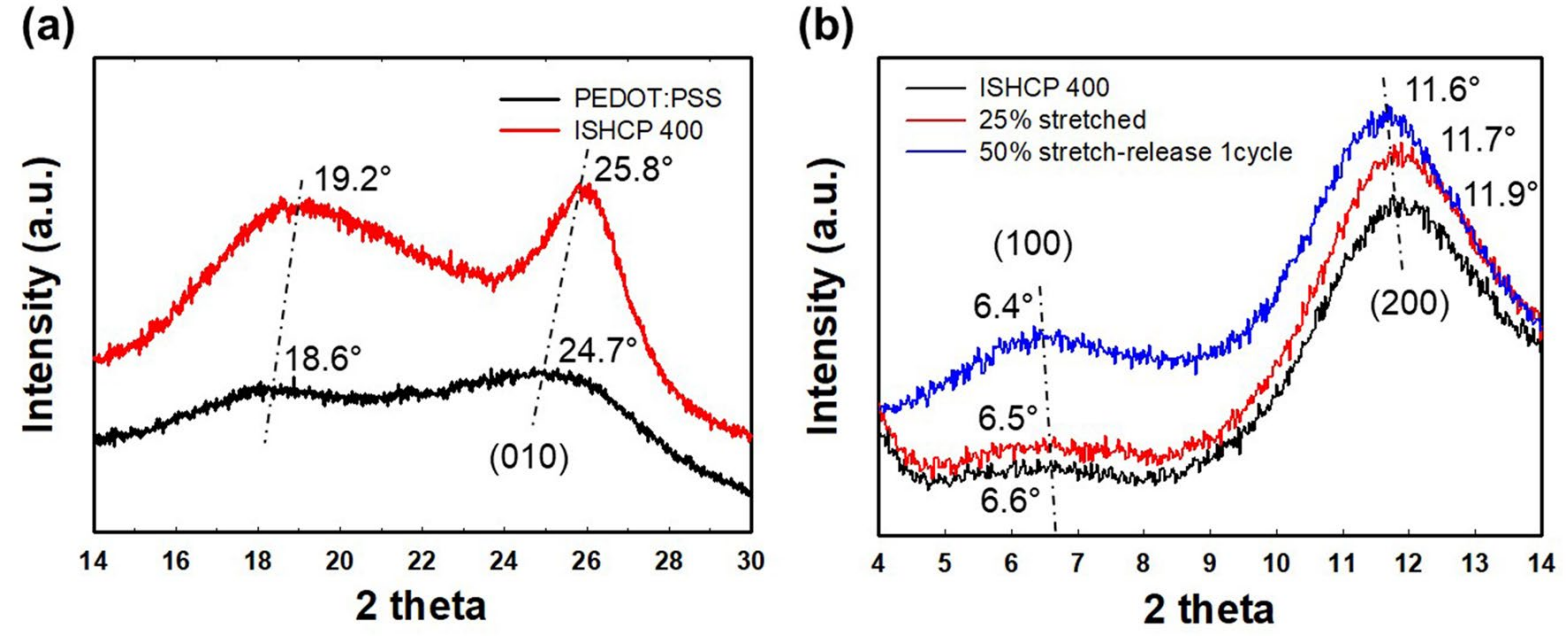
XRD spectra of PEDOT:PSS and ISHCP-400 for **(a)** freestanding sheets and **(b)** films coated on SEBS substrates under strain.

X-ray diffraction (XRD) analysis of freestanding sheets was conducted to measure the crystallinity change at the molecular level (Fig. 6a). For ISHCP-400 films, the intensity of peaks is stronger and sharper than that of neat PEDOT:PSS. In comparison with neat PEDOT:PSS, the characteristic two-theta peaks of ISHCP-400 showed a shift from 18.6° to 19.2° and from 24.7° to 25.8°. This phenomenon indicates reduced d_(010)_ spacing, which implies a decrease in the interchain π–π stacking distance of PEDOT and crystallization enhancement^28,29^. Conformational change of ISHCP-400 films under strain was observed on thin films coated on SEBS substrates (Fig. 6b). Compared to results before it was stretched, the peaks shifted to a lower angle in the 25% stretched state. The lower angle peak indicates broadened d_(100), (200)_ spacing intended to expand the coil structure. Linear lamellar stacking structure was formed by the unidirectional alignment of chains. Also, the films after one stretch-release cycle to 50% strain showed clearer peaks at a lower angle. It was assumed that the wrinkles formed by recovered ISHCP on SEBS substrates induced the shift of peaks.

**Figure 7:**
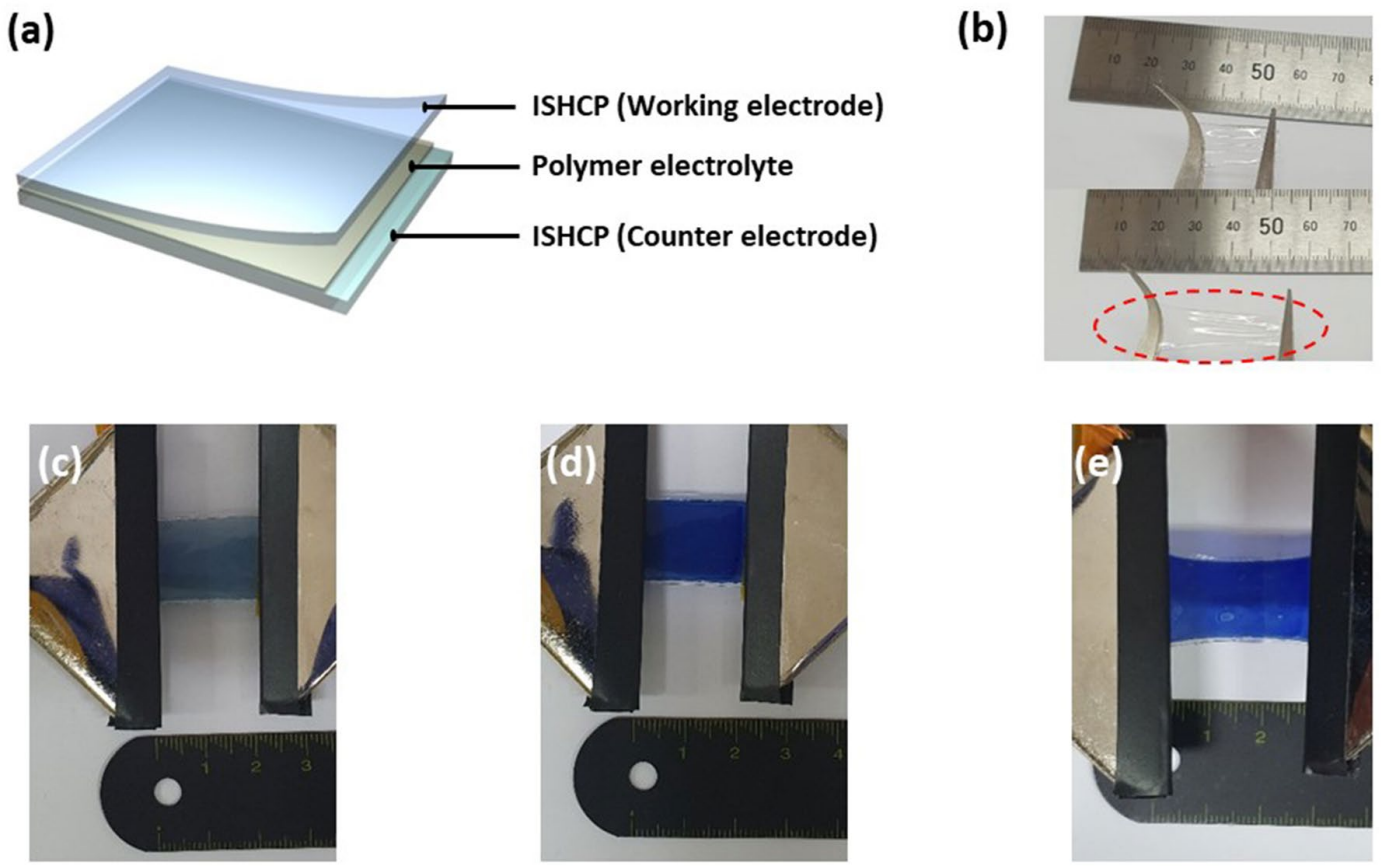
**(a)** Schematic for simple configuration of the stretchable ECDs. **(b)** Photograph of stretchable polymer electrolyte films. Photographs of **(c)** bleached, **(d)** colored, and **(e)** 50% stretched states of the stretchable ECD.

Fully Stretchable ECDs were fabricated using intrinsically stretchable and conductive EC polymers (ISHCP-400). In general, ECDs are fabricated by depositing EC materials onto a transparent electrode and placing an electrolyte between two electrochromic layers. To fabricate ideal stretchable ECDs, our simple configuration of the devices involved only two ISHCP-400 films and a stretchable electrolyte. As shown in Fig. 7a, the electrolyte layer was placed between the ISHCP films coated on the SEBS substrate. For our fully stretchable ECDs, ISHCP-400 can act as both the transparent electrode and the electrochromic layers at the same time. Hence, we fabricated the ideal, stretchable ECDs based on ISHCP with a simple device configuration, compared to typical electrochromic devices with five-layer configurations.

Moreover, the poly(acrylamide)-based hydrogel, containing *N,N,N´,N´*-tetramethyl-*p*-phenylenediamine (TMPD) as a polymer electrolyte, was introduced into the ECDs to provide excellent stretching characteristics and high transparency (91% at 550 nm) (Fig. 7b). Photo images of the stretchable ECDs based on ISHCP are shown in Fig. 7c, d. When a sufficient electrical potential (− 1.2 V) is applied, the stretchable ECDs in the bleached state change color from light blue to a dark cobalt blue. Figure 7e shows that the colored state of the stretchable ECDs maintained at a 50% strain state.

To determine the redox reaction of ISHCP and TMPD, cyclic voltammetry analysis was conducted. Electrochromic properties of the ISHCP film are similar to those of the PEDOT:PSS film. The ISHCP films in the ECDs have the color-changing property that is effected either by an electron-transfer (redox) process or by a sufficient electrochemical potential. The ISHCP film is light blue when all PEDOT units are in the oxidation state (PEDOT^-^). The ISHCP film changes to a dark blue color when PEDOT^0^ units are generated by electrochemical reduction. The surface redox chemistry is related to the phase behavior of PEDOT:PSS film described below:

1 $$\left[ {\left( {{\text{PEDOT}}^{ + } } \right)\left( {{\text{PSS}}^{ - } } \right) \, + {\text{ H}}^{ + } } \right]_{{{\text{bleached}}}} \leftrightarrow \left[ {{\text{PEDOT}}^{0} + \, \left( {{\text{PSS}}^{ - } } \right)\left( {{\text{H}}^{ + } } \right)} \right]_{{{\text{colored}}}}$$

**Figure 8:**
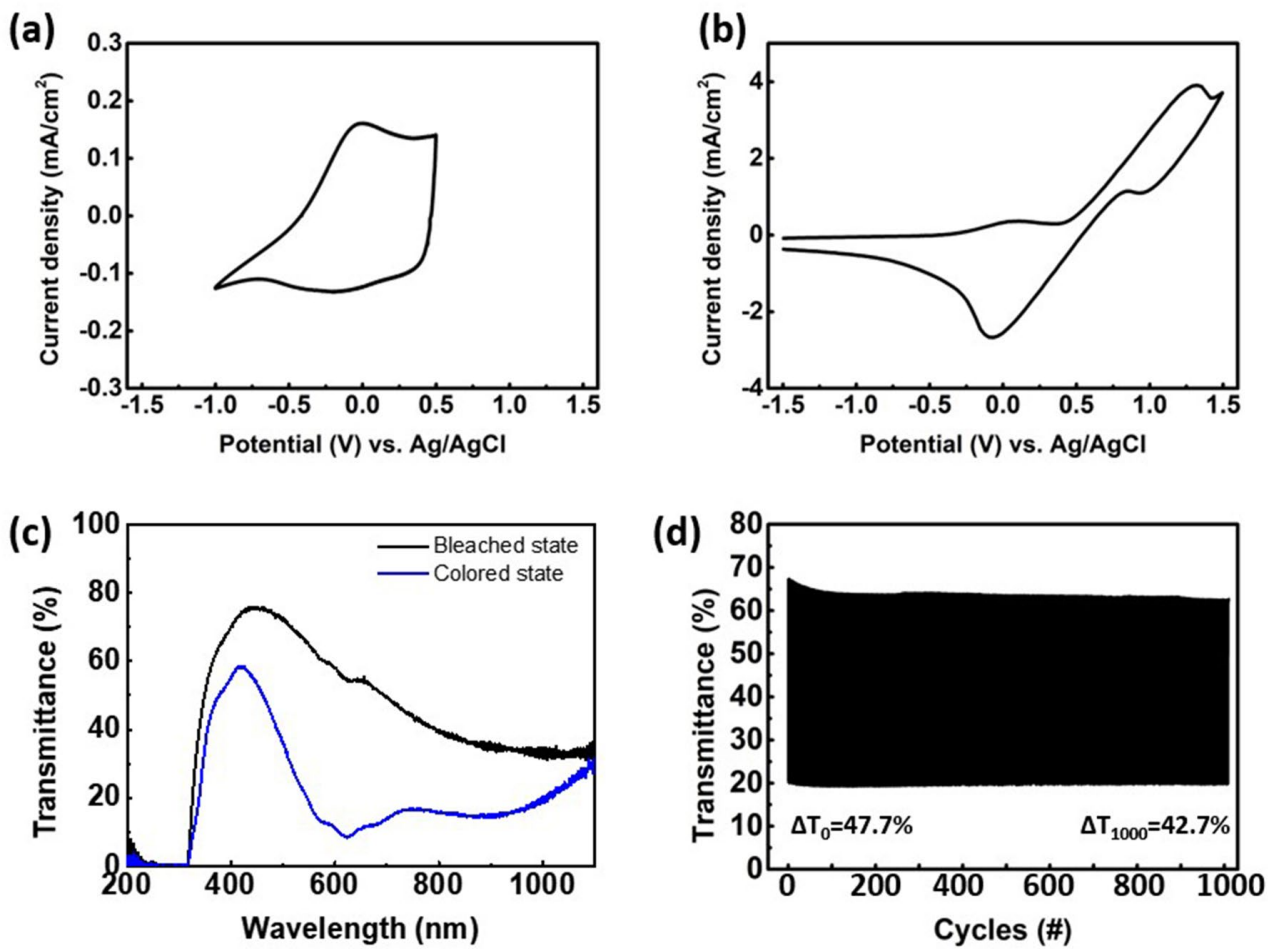
Cyclic voltammetry for ISHCP films in **(a)** 1 M H_2_SO_4_ and **(b)** 1 M H_2_SO_4_/0.1 M TMPD solution. Transmittance spectra of **(c)** the stretchable ECD at the bleached and colored states in the wavelength range of 180–1100 nm. **(d)** Cycle reliability over 1000 cycles of the stretchable ECD under − 1.2 V and 0 V at 590 nm.

For a complementary EC device, the reactions at each electrode must be balanced; that is, device performance is greatly affected by the charge capacity ratio of the two electrodes. We introduced TMPD as the anodic electrochromic material and ferrocene carboxylic acid (FcCOOH) as a redox mediator to the polymer electrolyte in the ECDs^30,31^. When an oxidation reaction occurs, TMPD change from colorless to cobalt blue color. Ferrocene carboxylic acid have good solubility in electrolyte solution, and allow a stable redox reaction with low potential due to presence of anodic species. Figure 8a, b shows the cyclic voltammograms (CV) of the ISHCP film in 1 M H_2_SO_4_ electrolyte solution and 1 M H_2_SO_4_ electrolyte solution containing 0.1 M TMPD, ferrocene carboxylic acid, at a scan rate of 50 mV s^−1^. For both electrolyte solutions, the potential of the relatively stable CV cycles was located between − 1.5 and 1.5 V. Two redox couples are clearly observed in Fig. 8b. The first redox couple, ranging from − 0.1 to 0.1 V, was contributed by the oxidation of FcCOOH to FcCOOH^+^, TMPD to TMPD^•+^, and PEDOT^0^ to PEDOT^+^ in the counter electrode and the reduction of PEDOT^+^ to PEDOT^0^ in the working electrode. The second redox peak, ranging from 0.9 to 1.2 V, is the oxidation of PEODT^0^ to PEDOT^+^ in the working electrode and the reduction of FcCOOH^+^ to FcCOOH, TMPD^•+^ to TMPD, and PEDOT^+^ to PEDOT^0^ in the counter electrode.

We also determined optical properties of ECD for different applied voltage. The UV–VIS transmittance spectra of the ISHCP films for 1 M H_2_SO_4_ electrolyte and 1 M H_2_SO_4_/0.1 M TMPD electrolyte are shown in Fig. S5. The transmittance spectra of the stretchable ECDs were taken in bleached, and different colored states when − 0.2, − 0.4, − 0.6, − 0.8, − 1.0, and − 1.2 V were applied. The maximum optical modulation (∆T) of the stretchable ECD containing TMPD is obtained at an applied potential − 1.2 V. The sufficient applied potential is lower compared with the pristine ISHCP film (− 1.8 V). TMPD has its characteristic peaks at 590 nm and 645 nm from 0 to − 1.2 V^32,33^. Figure 8c shows high optical modulation (47.7% at 590 nm) of the stretchable ECD containing TMPD between 0 and − 1.2 V. These results are evidence that H^+^ ion extraction was more effective due to diffusion of TMPD^•+^ in the polymer electrolyte. Furthermore, good charge balance is responsible for electrochemical stability of ECD by introducing TMPD as anodic electrochromic material. The CV analysis of the ECDs was periodically measured for ten cycles in a two-electrode system (Fig. S6). For the case of the ECD containing TMPD, there was no change in the redox behavior during the 10th cycle compared to pristine ECDs. These results revealed that the current density of the ECD containing TMPD only slightly decreases. As a result, TMPD^•+^ generated by electrochemical oxidation causes a more effective insertion/extraction of ions with opposite charges to balance the internally created electric fields. Thus, the balanced charge transport in the ECD containing TMPD significantly influences optical modulation and response speed^34,35^. In order to evaluate electrochemical durability of the ECD, cyclic stability was tested from − 1.2 V to 0 V at 590 nm (see Fig. 8d). Although the optical modulation (∆T) slightly decreased until initial 100 cycles, it showed good durability and maintained 42.7% at 1000th cycle. Thus, introduction TMPD into electrolyte enhanced the electrochromic cyclic reliability of the ECD.

**Figure 9:**
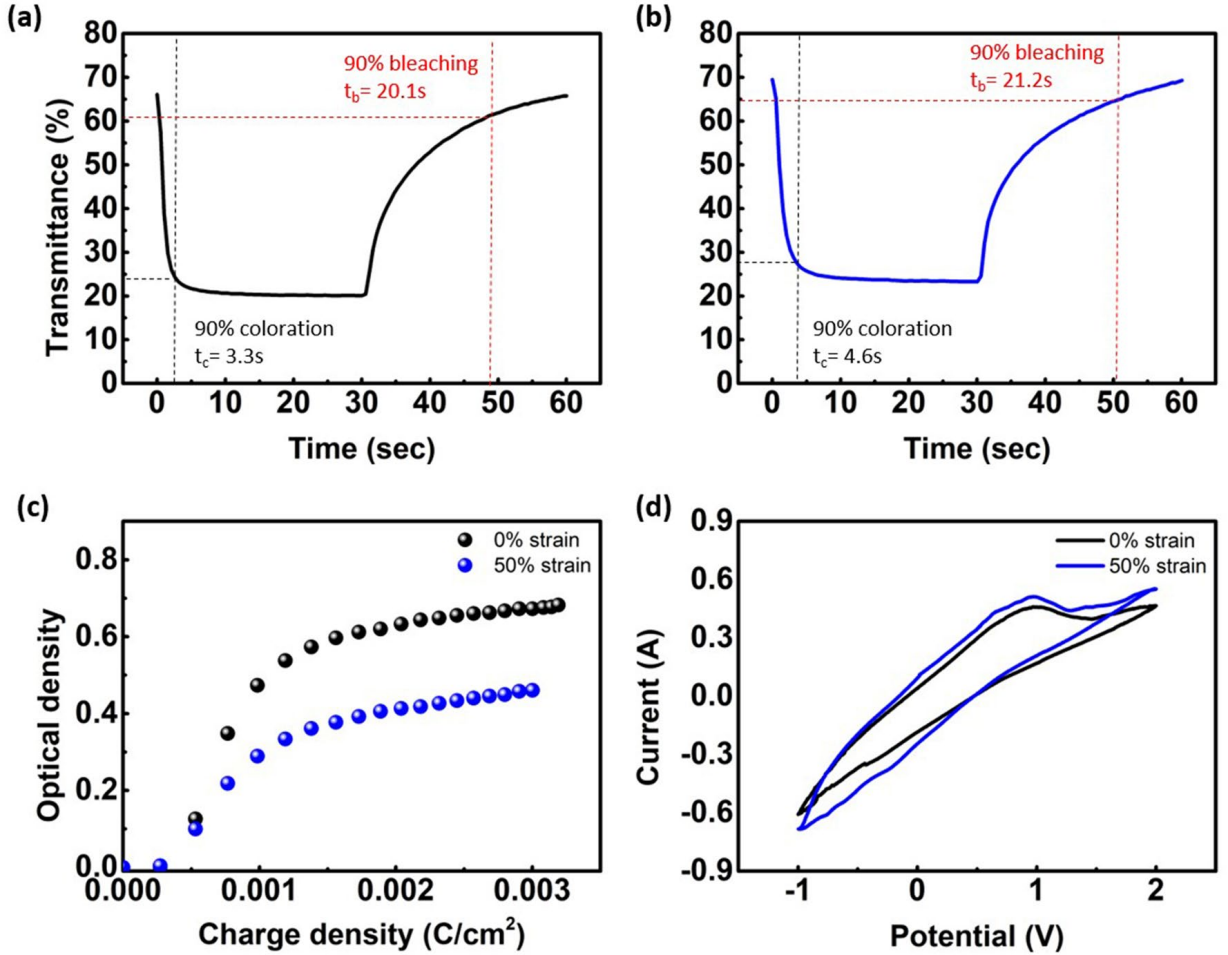
Coloration time and bleaching time of the stretchable ECD at **(a)** 0% strain and **(b)** 50% strain under applied potential − 1.2 V and 0 V with 30 s interval. **(c)** Coloration efficiency and **(d)** cyclic voltammetry curves of stretchable ECD at 0% and 50% strain.

To determine electrochromic properties of the fully stretchable ECDs, we obtained an in-situ optical response of the stretchable ECDs (Fig. 9a, b). All analyses for electrochromic properties were performed with the 15 mm $\times$ 20 mm size electrochromic films by alternating between an operating voltage of − 1.2 V and an off state of 0 V for 30 s interval. We have defined t_c_ (coloration time) as the time when the color changes by 90% and t_b_ (bleaching time) as the time when it becomes 90% transparent. The stretchable ECDs exhibited a switching speed of 3.3 s/20.1 s for the coloration/bleaching process at the 0% strain state. In Fig. 9b, coloration time is 4.6 s, and bleaching time is 21.2 s at the 50% stretched state. Here, the coloration time and the bleaching time are longer than the 0% stretched ECD. The optical modulation of 50% stretched ECDs is 45.2% at 590 nm. As noted in Fig. 2, this result may be due to the increasing sheet resistance of the ISHCP film and polymer electrolyte as it is stretched. In addition, it is expected that the electrochromic properties were affected as the electrical network of the ISHCP film was rearranged.

In EC devices, charge balance is essential for high optical modulation and fast response. Moreover, it is possible to fabricate ECDs with fast response and low power consumption if they exhibit sufficiently high coloration efficiency^36^. The coloration efficiency (CE, η) is defined as the change of optical density (∆OD) per unit charge of insertion or extraction. It is calculated by the following equation:

2 $$\eta \left(\lambda \right)=\frac{\mathrm{\Delta OD}\left(\lambda \right)}{Q}={\mathrm{log}}_{10}(\frac{{T}_{bleach}\left(\lambda \right)}{{T}_{color}(\lambda )})/Q,$$

where ∆OD is the change in optical density, Q is the consumed electrical charge density (C/cm^2^), and T_bleach_ and T_color_ are the optical transmittances at a specific wavelength (λ_max_) in the bleached and colored states, respectively. We calculated coloration efficiency of the stretchable ECD at 90% of the full coloration. The CE values were 434.1 cm^2^/C at 0% strain and 232.4 cm^2^/C 50% strain (590 nm). Clearly, the coloration efficiency obtained from ISHCP is much higher than the coloration efficiency of inorganic EC materials. From Fig. 9c, it is evident that ISHCP exhibits good electrochromic performance. Furthermore, the stretchable ECD maintains stable electro-activity under 50% strain, as shown in Fig. 9d.

**Figure 10:**
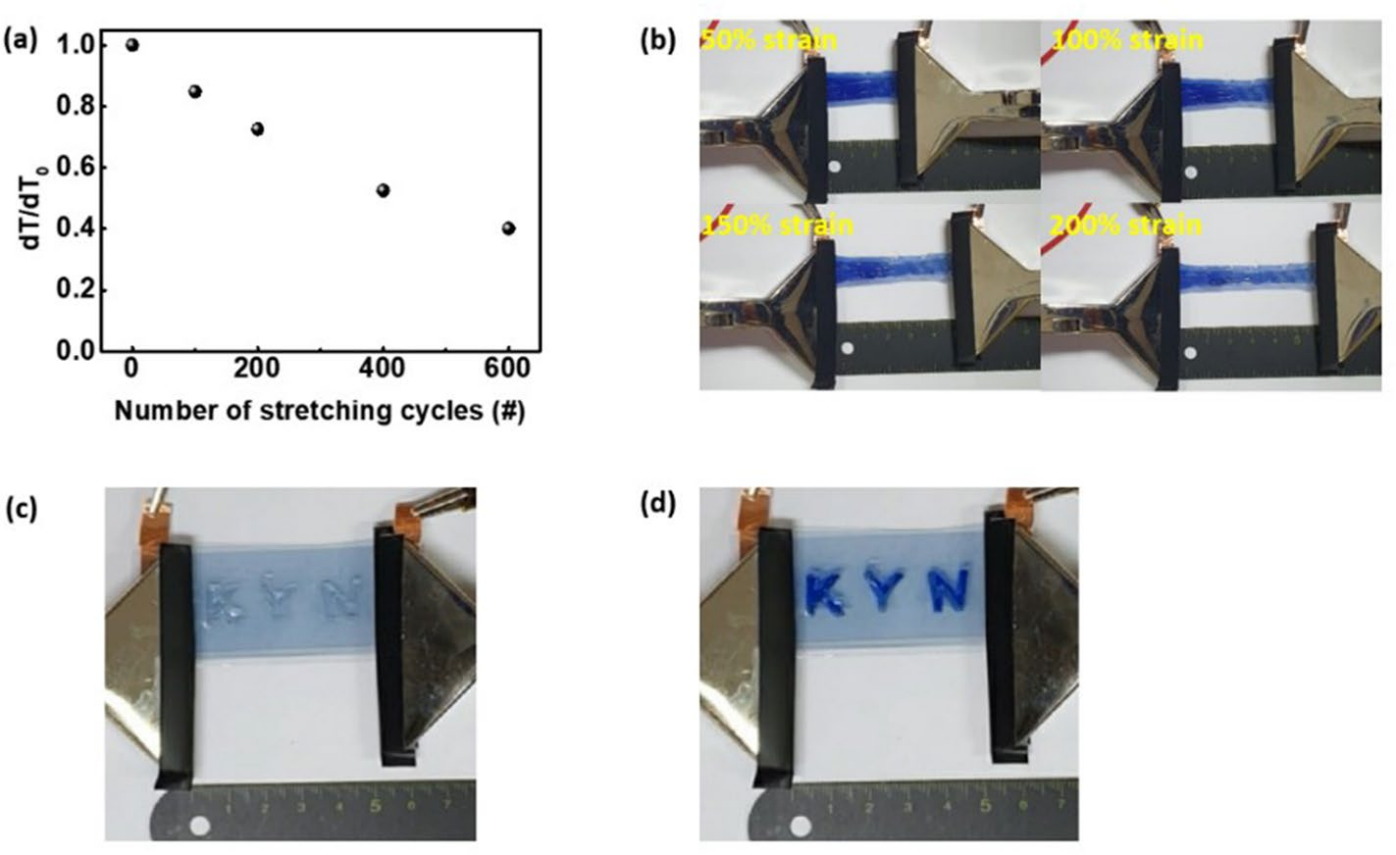
**(a)** Stretchable stability of the stretchable ECDs at the 50% strain state for up to 600 stretching cycles. **(b)** Photographs of the stretchable ECDs at 50%, 100%, 150%, and 200% strain. Patterned ECDs at the **(c)** bleached and **(d)** colored state (30 × 50 mm^2^ size).

The ISHCP was unidirectionally aligned at the stretched state and maintained not only electrical properties but also ion insertion and extraction of ECDs. Also, we conducted repeated stretching tests to evaluate the stability of the stretchable ECDs. Although the optical modulation decreased due to the harsh environment (50% strain repeat test), the stretchable ECD was operated reliably even after 600 cycles (Fig. 10a). Figure 10b shows photographs of the stretchable ECDs with − 1.2 V applied at 50%, 100%, 150%, and 200% strain states. Above the 100% strain state, the polymer electrolyte is found to have weaker stretchability than the ISHCP films, but EC performance of the ECDs is maintained even after stretching up to 200%. Also, the bending test compared to typical ITO/PET films was measured with change of relative sheet resistance of samples as shown in Fig. S7. More than 200 cycle of bending tests with 5 mm radius of curvature, the electrical properties of ITO/PET electrode and ECDs based on ITO electrode declined significantly. We fabricated the pattern “K, Y, N” on stretchable ECDs using a UV-curable polymer electrolyte. As shown in Fig. 10c, d, the patterned ECDs with -1.2 V applied exhibited excellent EC performance. The patterned ECDs worked very stably, even during the repeated stretching test on 100% strain (Supplementary Video 1). These results reveal that the stretchable ECDs based on ISHCP have promising potential for use in wearable electronic devices such as displays.

## Conclusions

We proposed a fully stretchable electronic device system consisting of ISHCP material that can simultaneously act as electrodes and EC layers. The semi-IPN between PSS and PEG, via sulfuric acid catalyst, exhibited good elasticity. The optimized, intrinsically stretchable ISHCP-400 films retained almost 100% of its electrical properties under 50% strain. The films exhibited the highest 1,406 S/cm electrical conductivity at 20% strain due to unidirectionally aligned molecular chains. The fabricated ECDs consisted of all-stretchable polymers, showing solution process-ability for large-scale production. The fully stretchable ECDs show the best performance of 47.7% ∆T, 434.1 cm^2^/C at 590 nm, and remained 45.2% ∆T after 50% strain stretching. Additionally, we realized a simple patterned electrolyte layer on our fully stretchable ECDs. Ultimately, the fully stretchable ECDs present potential applications in next-generation electronic devices, such as a stretchable display or smart window.

## Methods

Materials: 3,4-ethylenedioxythiophene (EDOT, ≥ 97%), poly(4-styrenesulfonic acid) (PSSA), iron(III) sulfate hydrate (Fe_2_(SO_4_)_3_, ≥ 97%), sodium persulfate (Na_2_S_2_O_8_, ≥ 99%), EG, poly(ethylene glycol) (PEG), toluene, SEBS, *N,N,N′,N*′-tetramethyl-*p*-phenylenediamine (TMPD, 99%), ferrocene carboxylic acid (FcCOOH, ≥ 97.0%), poly(ethylene glycol) diacrylate, and 1-hydroxycyclohexyl phenyl ketone (99%) were purchased from Sigma Aldrich Co., Yongin-Si, Gyeonggi-do, Korea. Sulfuric acid (H_2_SO_4_, 95%) was purchased from Duksan Pharmaceutical CO. (Seoul, Korea). All chemicals were used as received and without further purification. The ion (cation and anion) exchange resins were purchased from Samyang Co. Distilled deionized (DDI) water was used in all the experiments.

Synthesis of H_2_SO_4_ in-situ PEDOT:PSS and formulation for ISHCP: The polymerization via H_2_SO_4_ in-situ PEDOT:PSS was performed using sulfuric acid, which was 3 eq. molar ratio of the EDOT monomer. After the sulfuric acid and poly(sodium-4-styrenesulfonate) (PSS) were dissolved in DI-water, the remainder of the EDOT, Fe_2_(SO_4_)_3_ and Na_2_S_2_O_8_ were injected into the reactor. The reaction temperature was 10 ℃, and the conditions were kept constant for 24 h under an argon atmosphere. The synthesis followed the Baytron P procedure, and the ratio of PEDOT to PSS was 1:2.5 wt%^37^. After the reaction time of 24 h, cation and anion exchange resin was added to the reactor to remove the residual initiator and any other impurities of the reactions, such as sodium salt, and stirred for 2 h. The solid contents of the acquired solution were adjusted to 1.0 wt%. After homogenization of the solution, a dark blue liquid solution was obtained H_2_SO_4_ in-situ PEDOT:PSS. The H_2_SO_4_ in-situ PEDOT:PSS solution was treated by 7% EG and 1% poly(ethylene glycol) (PEG) with different molecular weights from 400 to 200,000 and a wetting agent to improve electrical conductivity, mechanical stretchability and coating ability.

Fabrication of ISHCP thin films on SEBS substrates: SEBS resin of 20 wt% was dissolved in toluene and stirred for 1 h. BYK-333 was added at 0.05 wt% for coating uniformity. The SEBS solution was coated by a doctor blade coater on liquid crystal display glass, with a size of 150 mm × 150 mm, to a wet thickness of 500 µm. The films were first dried at 60 ℃ for 10 min and then at 100 ℃ for 10 min. The measured thickness of the fabricated SEBS substrate was 50 µm. The above-completed ISHCP or PEDOT:PSS solution was bar coated with the desired thickness on SEBS substrates and dried at 150 ℃ for 10 min.

Fabrication of freestanding ISHCP sheets: In order to obtain freestanding sheets without a substrate, the ISHCP solution filled in a petri-dish was dried at 120℃ in a convection oven overnight. The sample was left at room temperature for 1 h and obtained by peeling the freestanding sheet off the petri dish.

Fabrication of polymer electrolyte and stretchable ECDs: ECDs based on ISHCP were fabricated by laminating working electrode and counter electrode coated ISHCP on a SEBS substrate. Before the laminating process, a polymer electrolyte layer was coated on the working electrode using the doctor-blade method. The polymer electrolyte solution was composed of 1 M H_2_SO_4_, acrylamide based UV-curable resin, and contained 0.1 M TMPD. The UV resin solution was prepared by placing 8 g of acrylamide and 10 mL of distilled water in a stirred system at room temperature for 1 h. After stirring, 0.78 g of PEGDA and 0.12 g of photoinitiator-184 were added to the acrylamide solution. The electrolyte solution was prepared by adding 0.25 g of TMPD and 0.02 g of FcCOOH to 1 M H_2_SO_4_ solution with stirring. Finally, the electrolyte solution was mixed with the UV resin solution in a 1:1 ratio at room temperature for 2 h. The polymer electrolyte was cured using UV light (wavelength equal to 365 nm) for 20 s. Copper tape (or silver paste) was used for making contacts with the electrodes of the EC film and for preventing voltage drop. All of the stretchable ECDs were fabricated to a size of 15 mm × 20 mm (width × length).

Sample Characterization: To measure the electrical conductivity of the thin films, sheet resistance was measured by a four-point probe system (Napson RT-70). A digital multimeter was used to observe the change of thin-film resistance under strain. The mechanical stretchability and Yong’s modulus was measured by a universal testing machine using freestanding sheets of PEDOT:PSS and ISHCP. The surface of the thin films was observed by scanning electron microscopy (JEOL-7610F-Plus) and atomic force microscopy (XE-100, Park Systems) with topographic and phase images. The crystallinity and conformational change of films were measured by XRD (SmartLab, Rigaku). Electrochemical measurements were performed in a three-electrode cell with ISHCP films as the working and counter electrodes and Ag wire as the reference electrode. The voltage was controlled using an AMETEK PARSTAT MC potentiostat/galvanostat. The transmittance of the stretchable ECDs was measured by a AVATES UV–VIS spectrometer.

## Supplementary information

Supplementary Information.

Supplementary Video.

## Data Availability

*Scientific Reports* requires the inclusion of a data availability statement with all submitted manuscripts, as this journal requires authors to make available materials, data, and associated protocols to readers.
