# Supplementary material for: Design of intrinsically stretchable and highly conductive polymers for fully stretchable electrochromic devices
Source: Sci Rep. 2020 Oct 5;10:16488. doi: 10.1038/s41598-020-73259-x (PMC7536397; doi:10.1038/s41598-020-73259-x)
Supplement: Supplementary file 1 — Supplementary Information. [file 41598_2020_73259_MOESM1_ESM.docx]

Supporting Information

Design of Intrinsically Stretchable and Highly Conductive Polymers for Fully Stretchable Electrochromic Devices

Youngno Kim^1⸸^, Chanil Park^1⸸^, Soeun Im^1^, and Jung Hyun Kim^1^*

^1^Yonsei University, Department of Chemical and Biomolecular Engineering, Seoul, 03722, South Korea ^*^jayhkim@yonsei.ac.kr
^⸸^these authors contributed equally to this work.


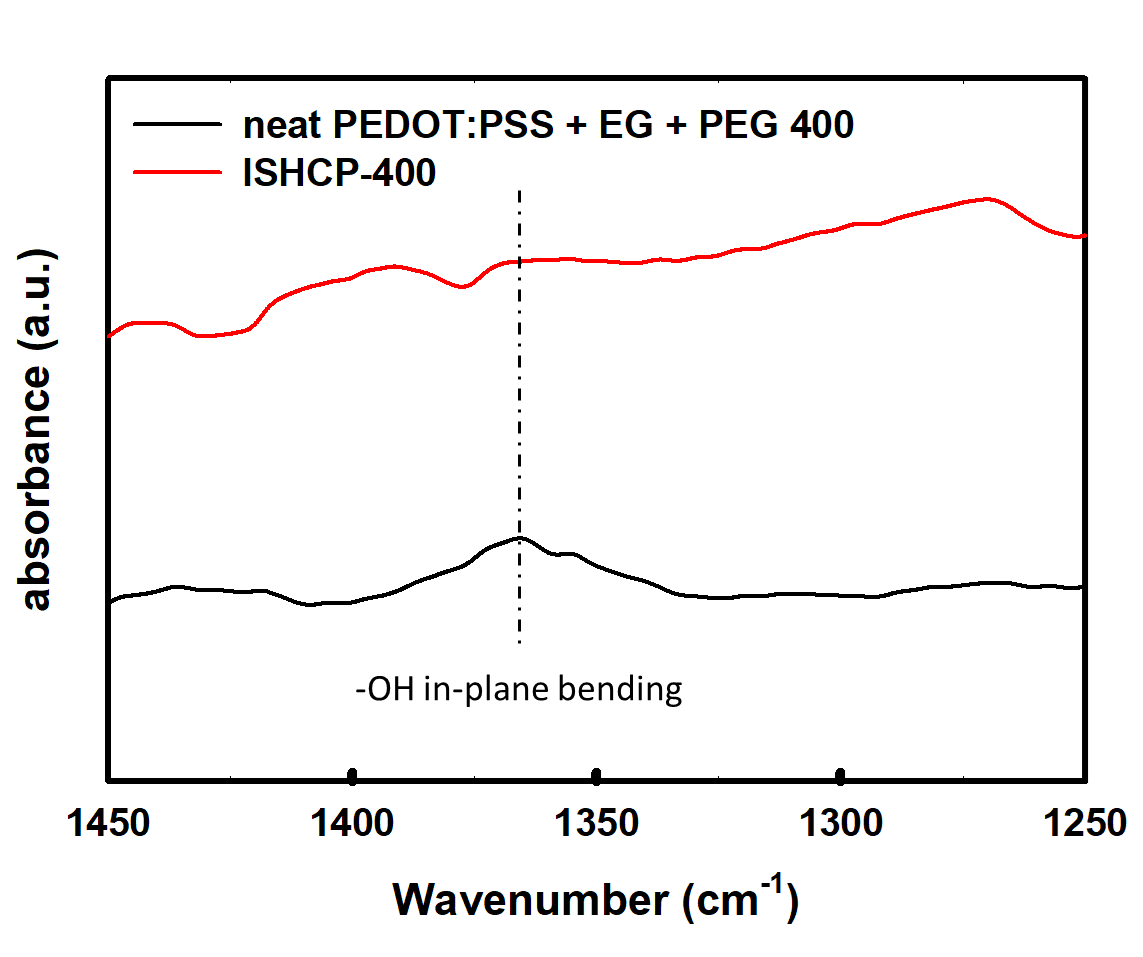


**Figure S1.** ATR-FTIR of freestanding films of PEDOT:PSS and ISHCP-400; hydroxyl group in-plane bending peak is indicated by the dashed line.


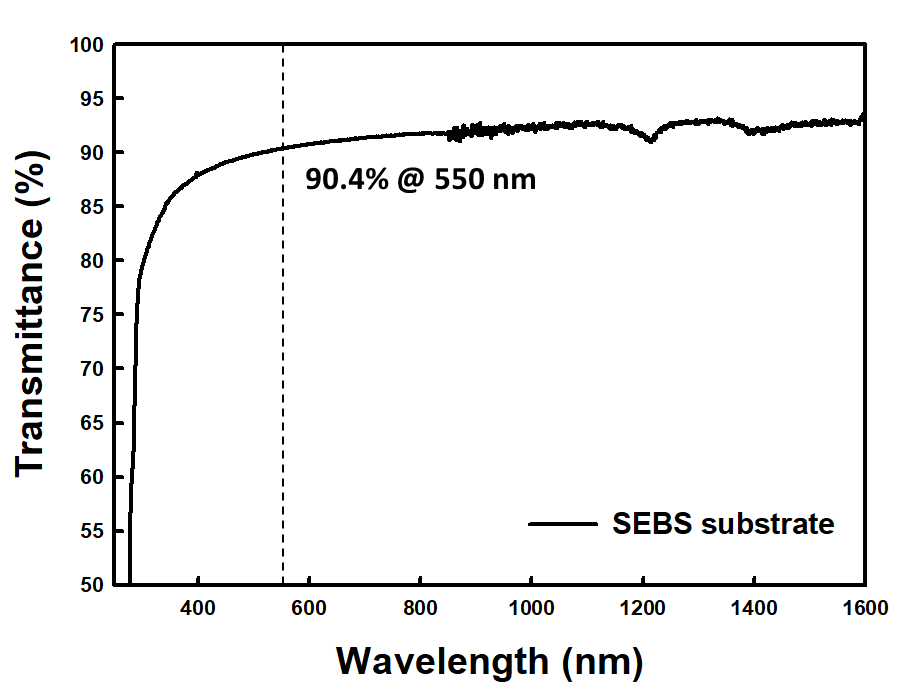


**Figure S2.** UV-Vis-NIR transmittance spectra of SEBS substrate on wavelength 250 ~ 1600 nm.


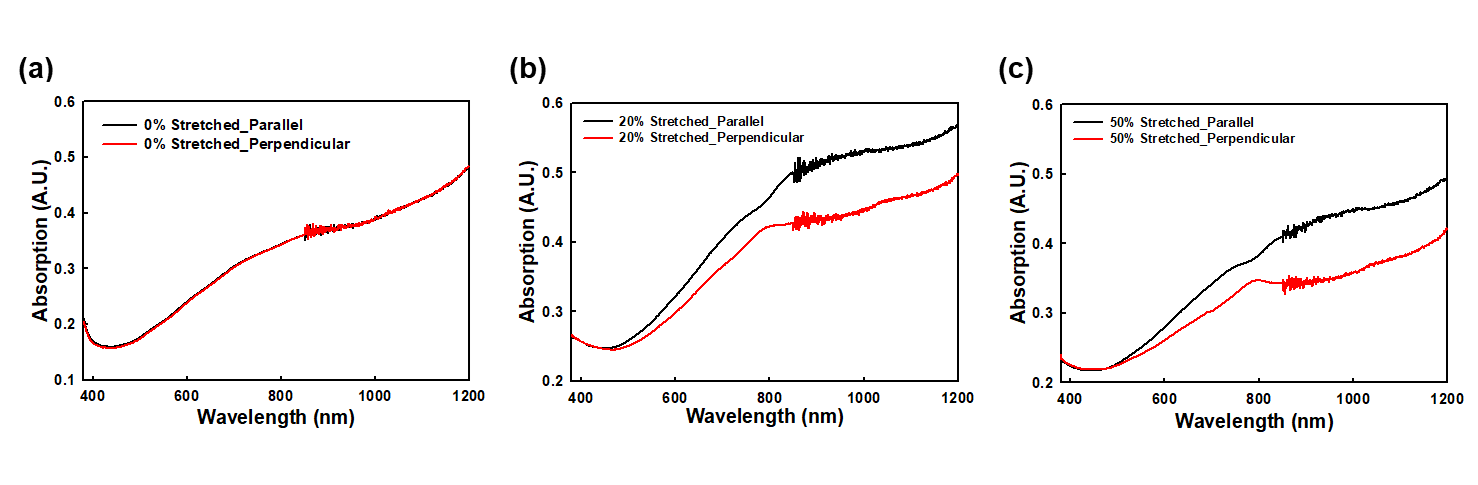


**Figure S3.** Polarized UV-Vis-NIR spectra of ISHCP-400 film under (a) 0%, (b) 20%, and (c) 50% tensile strains with parallel and perpendicular with respect to the stretching direction.


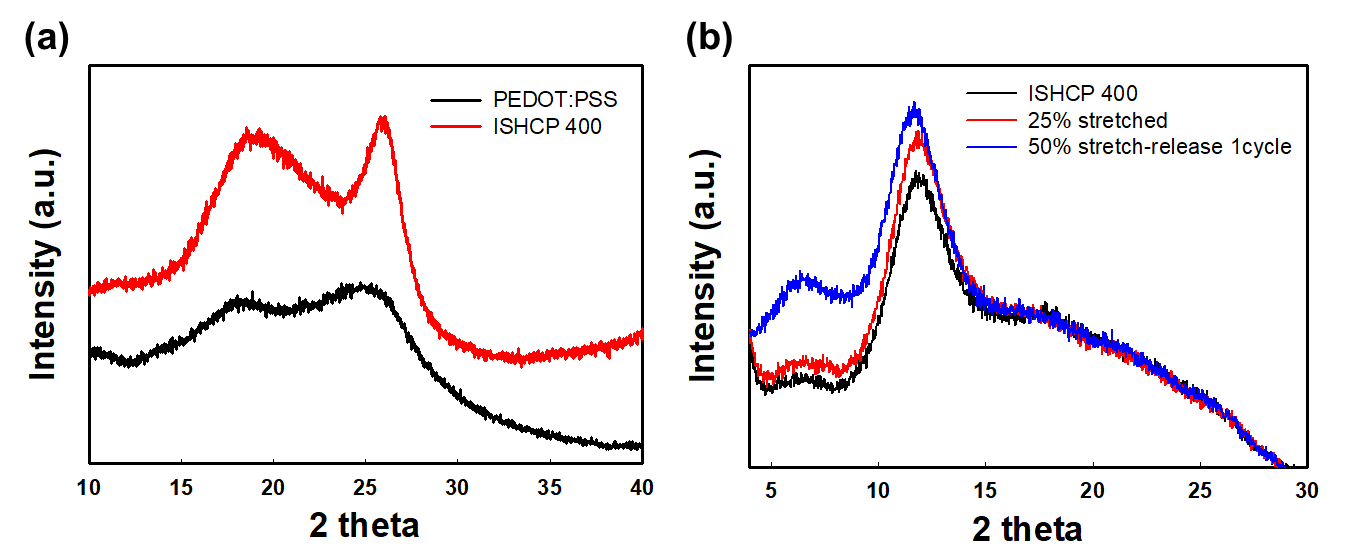


**Figure S4.** XRD spectra in a large range of PEDOT:PSS and ISHCP-400 for (a) freestanding film
and (b) films coated on SEBS substrates under strain.


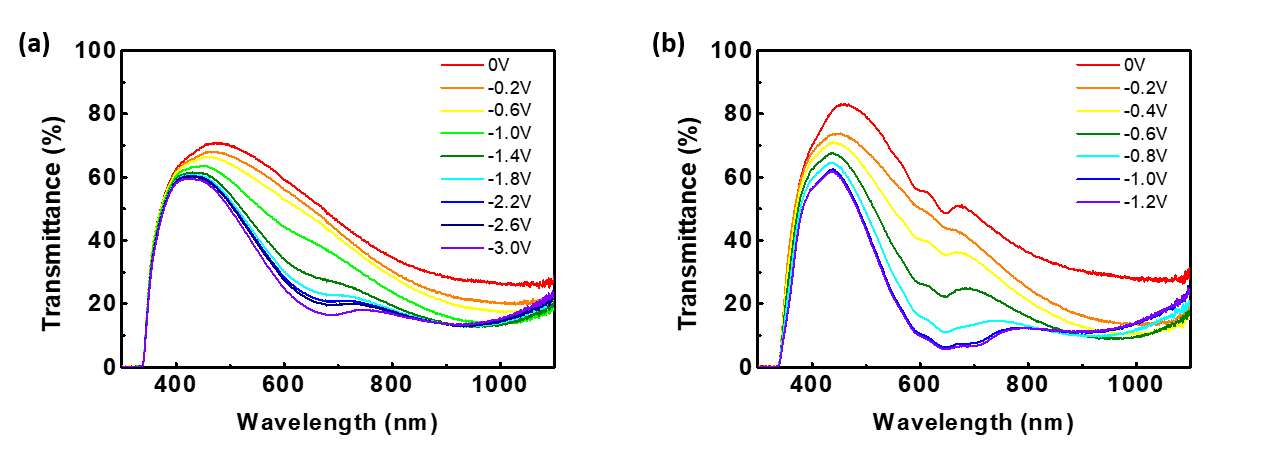


**Figure S5.** UV-vis transmittance spectra of ISHCP films for (a) 1M H_2_SO_4_ electrolyte and (b) 1M H_2_SO_4_ /0.1M TMPD electrolyte at various potential biases.


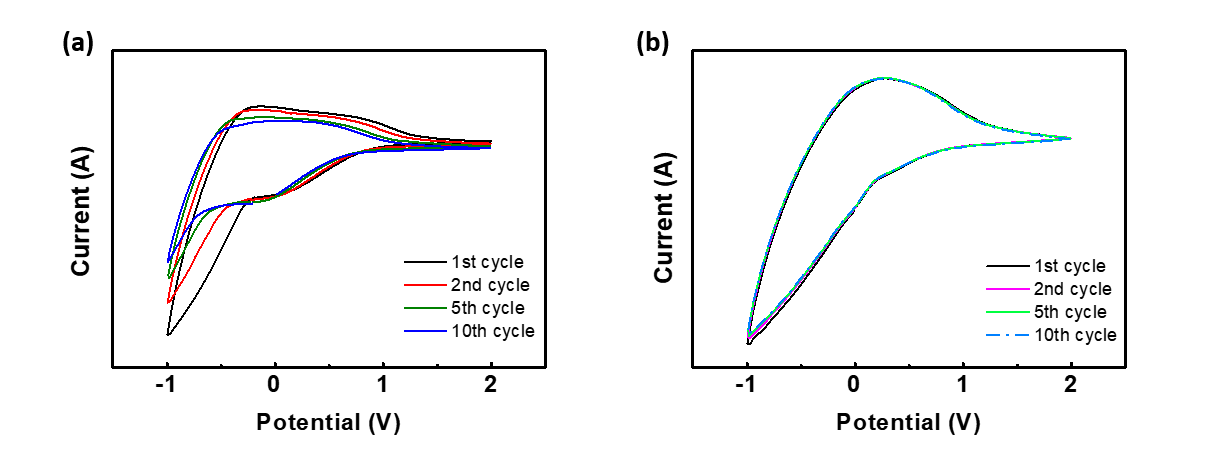


**Figure S6.** Electrochemical switching on lifetime of ECDs; (a) pristine ECDs based only on ISHCP and (b) ECDs containing TMPD.


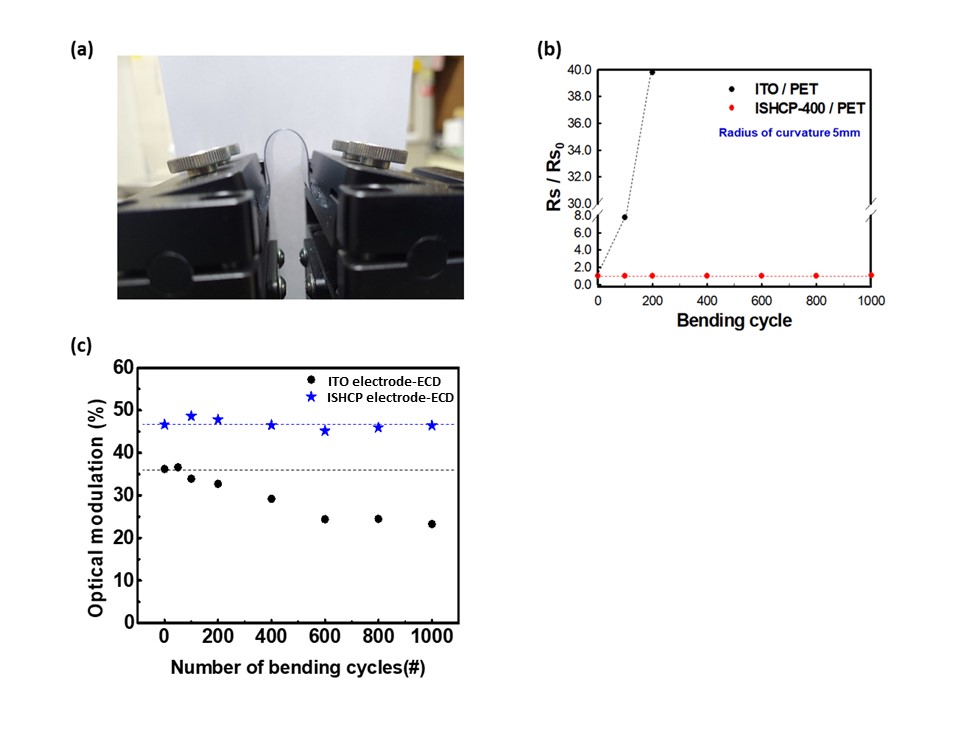


**Figure S7.** (a) Photograph of the bending test of the ECD banded on ISHCP. (b) Change of relative sheet resistance (*Rs/Rs_0_*) of ITO/PET and ISHCP-400/PET sheets under the cyclic bending test. (c) Optical modulation of the ECDs for up to 1000 bending cycles.


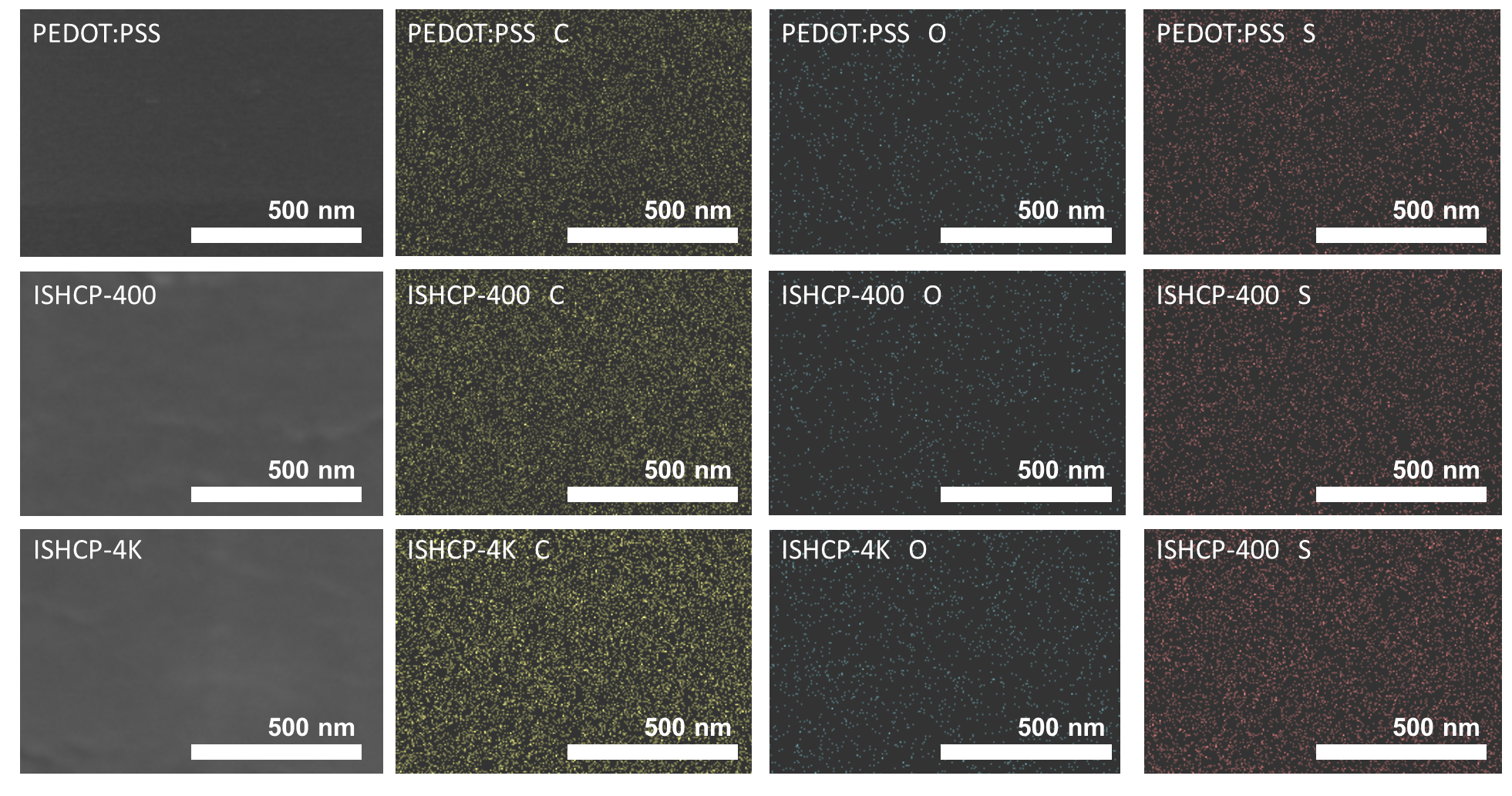


**Figure S8.** (a) SEM images and EDX mapping showing the distribution of carbon, oxygen and sulfur atom in the PEDOT:PSS and ISHCP layers.

**Table S1.** Measured sheet resistance, film thickness, and electrical conductivity of PEDOT:PSS and ISHCP materials with different additives.

| Material | Sheet resistance  [Ω/sq] | Film thickness  [nm] | Electrical conductivity  [S/cm] |
| --- | --- | --- | --- |
| PEDOT:PSS + PEG 400 | 146.8 | 524 | 130 |
| PEDOT:PSS + EG + PEG 400 | 83.9 | 461 | 258 |
| In-situ H_2_SO_4_ PEDOT:PSS + EG | 54.7 | 265 | 690 |
| In-situ H_2_SO_4_ PEDOT:PSS + EG + PEG 400 (ISHCP-400) ^a)^ | 38.7 | 289 | 891 |
| In-situ H_2_SO_4_ PEDOT:PSS + EG + PEG 600 (ISHCP-600) | 55.5 | 329 | 548 |
| In-situ H_2_SO_4_ PEDOT:PSS + EG + PEG 1K (ISHCP-1K) | 59.4 | 341 | 494 |
| In-situ H_2_SO_4_ PEDOT:PSS + EG + PEG 4K (ISHCP-4K) | 61.2 | 344 | 475 |
| In-situ H_2_SO_4_ PEDOT:PSS + EG + PEG 200K (ISHCP-200K) | 70.9 | 358 | 394 |

^a)^(the optimized recipe with best performing on electrical and deformable properties)

**Table S2.** Summary of performance of stretchable electrochromic device recently reported in the literature.

| Sample | Transparent electrode | Electrochromic materials | Elastomeric  substrate | Sheet resistance of  stretchable transparent electrode (Ω/□) | | Transmittance | Stretchability  of device |
| --- | --- | --- | --- | --- | --- | --- | --- |
|  |  |  |  | (at 0% strain) | (under stretched) |  |  |
| This work | ISHCP 400 | ISHCP 400 | SEBS | 38.7  (891 S/cm) | 41.1  (at 50% strain) | 77%  (at 550 nm) | 100% |
| [Ref 7]  2017,  Kai et al | PEDOT/PU | PEDOT/PU | PU | -  (10 S/cm) | - | - | 50% |
| [Ref 8]  2019,  Yun et al | Au/Ag NW embedded PDMS  WO_3_ nanotube / PEDOT:PSS | WO_3_ nanotube / PEDOT:PSS | PDMS | - | -  (at 40% strain) | 80% (at 635 nm) | 20% |
| [Ref 11]  2019,  Yang et al | Ag/PDMS | Polyacrylamide hydrogel with EC molecules | PDMS | 10 | 350  (at 30% strain) | 80%  (at 550 nm) | 20% |
| [Ref 10]  2018,  Lee et al | AgNW/PDMS | Viologen/PVA | PDMS | 36.2 | -  (at 50% strain) | 80%  (at 550 nm) | 20% |


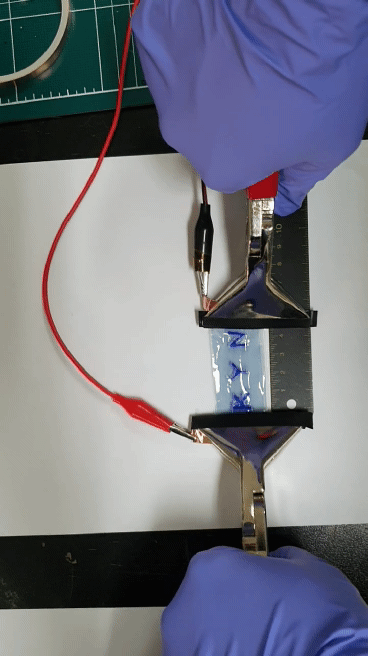


**Video S1.** Movie of cyclic stretch-release test with patterned electrochromic device from 0 to 100% strain.
