# Supplementary figures and images for: Design of intrinsically stretchable and highly conductive polymers for fully stretchable electrochromic devices
Source: Sci Rep. 2020 Oct 5;10:16488. doi: 10.1038/s41598-020-73259-x (PMC7536397; doi:10.1038/s41598-020-73259-x)

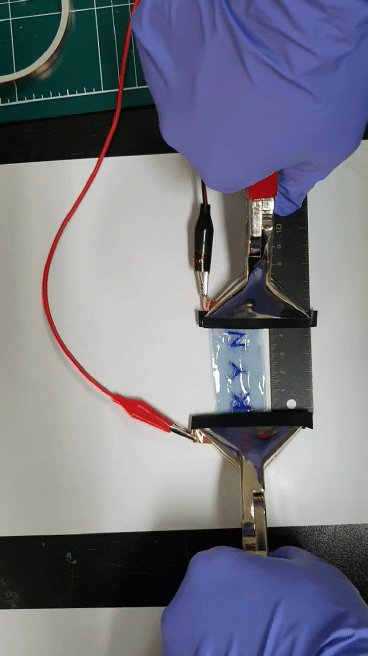

Supplement: Supplementary file 2 — Supplementary Video. [file 41598_2020_73259_MOESM2_ESM.gif]
